# Defining the Genetic Features of O-Antigen Biosynthesis Gene Cluster and Performance of an O-Antigen Serotyping Scheme for *Escherichia albertii*

**DOI:** 10.3389/fmicb.2017.01857

**Published:** 2017-09-26

**Authors:** Hong Wang, Han Zheng, Qun Li, Yanmei Xu, Jianping Wang, Pengcheng Du, Xinqiong Li, Xiang Liu, Ling Zhang, Nianli Zou, Guodong Yan, Zhengdong Zhang, Huaiqi Jing, Jianguo Xu, Yanwen Xiong

**Affiliations:** ^1^Zigong Center for Disease Control and Prevention, Zigong, China; ^2^State Key Laboratory of Infectious Disease Prevention and Control, Collaborative Innovation Center for Diagnosis and Treatment of Infectious Diseases, National Institute for Communicable Disease Control and Prevention, Chinese Center for Disease Control and Prevention, Beijing, China; ^3^Beijing Key Laboratory of Emerging Infectious Diseases, Institute of Infectious Diseases, Beijing Ditan Hospital, Capital Medical University, Beijing, China

**Keywords:** *Escherichia albertii*, O-AGC, serotype, antiserum, xTAG Luminex

## Abstract

*Escherichia albertii* is a newly described and emerging diarrheagenic pathogen responsible for outbreaks of gastroenteritis. Serotyping plays an important role in diagnosis and epidemiological studies for pathogens of public health importance. The diversity of O-antigen biosynthesis gene clusters (O-AGCs) provides the primary basis for serotyping. However, little is known about the distribution and diversity of O-AGCs of *E. albertii* strains. Here, we presented a complete sequence set for the O-AGCs from 52 *E. albertii* strains and identified seven distinct O-AGCs. Six of these were also found in 15 genomes of *E. albertii* strains deposited in the public database. Possession of *wzy*/*wzx* genes in each O-AGC strongly suggest that O-antigens of *E. albertii* were synthesized by the Wzx/Wzy-dependent pathway. Furthermore, we performed an O-antigen serotyping scheme for *E. albertii* based on specific antisera against seven O-antigens and a high throughput xTAG Luminex assay to simultaneously detect seven O-AGCs. Both methods accurately identified serotypes of 64 tested *E. albertii* strains. Our data revealed the high-level diversity of O-AGCs in *E. albertii*. We also provide valuable methods to reliably identify and serotype this bacterium.

## Introduction

Lipopolysaccharide (LPS) molecules form the outer leaflet of the outer membrane of many Gram-negative bacteria and are essential components of the bacterial cell envelope. The O-antigen polysaccharide constitutes the exterior part of the LPS and consists of oligosaccharide repeats (O-units) containing three to six sugar residues. The O-antigen plays an important role in resistance to phagocytosis and complement-mediate lytic action (Murray et al., 2003, 2006; Duerr et al., 2009; Saldias et al., 2009). Meanwhile, the O-antigen is a major surface antigen and is responsible for serological diversity of Gram-negative bacteria which are clinically and epidemiologically important to classify various strains. O-antigen has also provided a basis for development of vaccine against many pathogens. The genes required for O-antigen biosynthesis are clustered at a chromosomal locus, named the O-antigen biosynthesis gene cluster (O-AGC) in many bacteria. Generally, the genes in O-AGC are clustered into three major classes: sugar synthesis genes, glycosyltransferase genes, and O-unit processing genes. Polymerization of the O-units into an O-antigen is mostly mediated though two of three pathways in Gram-negative species: Wzx/Wzy-dependent pathway and ABC transporter-dependent pathway (Valvano, 2003). Synthase-dependent pathway, the third pathway, is rarely present in Gram-negative species. O-AGC is always located between the conserved *galF* (encoding UTP-glucose-1-phosphate uridylyltransferase) and *gnd* (encoding 6-phosphogluconate dehydrogenase) genes in many species of the *Enterobacteriaceae*, such as *E. coli* (Iguchi et al., 2015) and *Cronobacter sakazakii* (Mullane et al., 2008). Two genes, encoding the O antigen flippase (*wzx*) and O antigen polymerase (*wzy*), are unique in most of the O-AGCs, and have been used as targets for molecular O serogrouping (DebRoy et al., 2016).

*Escherichia albertii* is a newly described and emerging diarrheagenic pathogen, which is associated with both sporadic infections and outbreaks in humans (Ooka et al., 2013; Asoshima et al., 2014; Murakami et al., 2014; Brandal et al., 2015; Inglis et al., 2015). It was initially identified as *Hafnia alvei* and later proposed as *E. albertii*, a new species within the genus *Escherichia* (Huys et al., 2003). *E. albertii* strains were often misidentified as *E. coli, Hafnia, Salmonella*, or *Yersinia ruckeri* as the lack of specific biochemical characteristics (Abbott et al., 2003). Thus, the prevalence of *E. albertii* may be underestimated owing to the lack of effective methods to discriminate *E. albertii* from other members of the *Enterobacteriaceae*. To date, little information on the *E. albertii* O- antigen is available. Only several chemical structures of the O-specific polysaccharide (OPS) of *E. albertii* were reported in previous study (Eserstam et al., 2002; Naumenko et al., 2017; Zheng et al., 2017). There is no comprehensive scheme for O-antigen classification of *E. albertii*. This study was aimed to investigate the prevalence and characteristics of O-AGCs in *E. albertii* strains and develop an O-antigen serotyping scheme and a high throughput detection assay to simultaneously detect all types of these O-AGCs.

## Materials and methods

### Bacterial strains and genomic DNA preparation

Fifty-two strains were selected in the current study: type strain LMG20976 (Huys et al., 2003); one strain from the stool of a diarrheal patient resident in Shanghai in 2013; and 50 strains isolated from multiple sources in Zigong city of Sichuan province between 2014 and 2015 (Table 1). Thirty of these were also used in our previous study (Wang H. et al., 2016). An additional 12 strains were isolated from Luzhou city of Sichuan province in 2016 and used in an agglutination test and the development of the high throughput xTAG Luminex detection assay (Table 1). Strains were cultured on Luria-Bertani (LB) plates (Oxoid, UK) and genomic DNA was extracted using the Wizard Genomic DNA Purification kit (Promega, Madison, MI, USA). Seven housekeeping genes were used for multi-locus sequence typing (MLST) analysis according to the *E. coli* MLST website (http://mlst.warwick.ac.uk/mlst/dbs/Ecoli). *E. coli* O3 and O181 antisera were purchased from Statens Serum Institut (SSI, Copenhagen, Denmark) for the agglutination test. All strains were verified to be *E. albertii* based on the combination of 16S rDNA sequencing, diagnostic multiplex PCR, and MLST analysis as described in our previous study (Wang H. et al., 2016).

**Table 1:** *E. albertii* strains used in this study.

**Strain**	**Source**	**Geography**	**Isolation time**	**Serotype**	**MLST sequence type (ST)**	**Accession number of O-AGCs**	**References**
LMG20976	Faece of diarrhoeal child	Dhaka, Bangladesh	90's	O7	ST383	KY574555	Wang H. et al., 2016
SP140128	Mutton	Zigong city, Sichuan province	2014	O1.1	ST4479	KY574559	Wang H. et al., 2016
SP140150	Chicken intestines	Zigong city, Sichuan province	2014	O1.2	ST4479	KY574562	Wang H. et al., 2016
SP140152	Chicken intestines	Zigong city, Sichuan province	2014	O2	ST3762	KY574563	Wang H. et al., 2016
SP140089	Chicken meat	Zigong city, Sichuan province	2014	O1.3	ST4479	KY574558	Wang H. et al., 2016
SP140149	Chicken intestines	Zigong city, Sichuan province	2014	O1.3	ST4479	KY574561	Wang H. et al., 2016
SP140148	Chicken intestines	Zigong city, Sichuan province	2014	O3	ST4633	KY574560	Wang H. et al., 2016
SP140047	Chicken intestines	Zigong city, Sichuan province	2014	O1.4	ST4479	KY574556	Wang H. et al., 2016
SP140084	Chicken meat	Zigong city, Sichuan province	2014	O3	ST4633	KY574557	Wang H. et al., 2016
SP140602	Chicken intestines	Zigong city, Sichuan province	2014	O4	ST4638	KY574564	Wang H. et al., 2016
SP140610	Chicken intestines	Zigong city, Sichuan province	2014	O3	ST4633	KY574565	Wang H. et al., 2016
SP140618	Chicken intestines	Zigong city, Sichuan province	2014	O2	ST3762	KY574566	Wang H. et al., 2016
SP140619	Chicken intestines	Zigong city, Sichuan province	2014	O5	ST1996	KY574567	Wang H. et al., 2016
SP140637	Chicken intestines	Zigong city, Sichuan province	2014	O4	ST4634	KY574568	Wang H. et al., 2016
SP140638	Chicken intestines	Zigong city, Sichuan province	2014	O4	ST4596	KY574569	Wang H. et al., 2016
SP140645	Chicken intestines	Zigong city, Sichuan province	2014	O4	ST4636	KY574570	Wang H. et al., 2016
SP140674	Chicken intestines	Zigong city, Sichuan province	2014	O6	ST4637	KY574571	Wang H. et al., 2016
SP140692	Duck meat	Zigong city, Sichuan province	2014	O2	ST3762	KY574572	Wang H. et al., 2016
SP140701	Chicken meat	Zigong city, Sichuan province	2014	O4	ST4638	KY574573	Wang H. et al., 2016
SP140724	Chicken intestines	Zigong city, Sichuan province	2014	O3	ST4633	KY574574	Wang H. et al., 2016
SP140733	Duck intestines	Zigong city, Sichuan province	2014	O4	ST4634	KY574575	Wang H. et al., 2016
SP140748	Duck intestines	Zigong city, Sichuan province	2014	O1.5	ST4479	KY596022	Wang H. et al., 2016
SP140749	Duck intestines	Zigong city, Sichuan province	2014	O1.6	ST4479	KY574576	Wang H. et al., 2016
SP140753	Duck intestines	Zigong city, Sichuan province	2014	O1.7	ST4479	KY574577	Wang H. et al., 2016
SP140754	Duck intestines	Zigong city, Sichuan province	2014	O1.4	ST4479	KY574578	Wang H. et al., 2016
SP140771	Chicken intestines	Zigong city, Sichuan province	2014	O3	ST4633	KY574579	Wang H. et al., 2016
SP140791	Pork meat	Zigong city, Sichuan province	2014	O5	ST4619	KY574580	Wang H. et al., 2016
SP140807	Chicken intestines	Zigong city, Sichuan province	2014	O4	ST4636	KY574581	Wang H. et al., 2016
SP140813	Chicken intestines	Zigong city, Sichuan province	2014	O3	ST4633	KY574582	Wang H. et al., 2016
SP140837	Duck intestines	Zigong city, Sichuan province	2014	O2	ST4639	KY574583	Wang H. et al., 2016
SP140839	Duck meat	Zigong city, Sichuan province	2014	O2	ST4639	KY574584	Wang H. et al., 2016
ZG141049	Diarrhea patient	Zigong city, Sichuan province	2014	O7	ST4947	KY574602	This study
ZG140067	Healthy human	Zigong city, Sichuan province	2014	O7	ST4947	KY574601	This study
D140513	Faece of egret	Zigong city, Sichuan province	2014	O4	ST4634	KY574553	This study
SP150020	Duck intestines	Zigong city, Sichuan province	2015	O2	ST3762	KY574585	This study
SP150021	Duck intestines	Zigong city, Sichuan province	2015	O2	ST3762	KY574586	This study
SP150027	Duck intestines	Zigong city, Sichuan province	2015	O2	ST3762	KY574587	This study
SP150036	Duck intestines	Zigong city, Sichuan province	2015	O5	ST1996	KY574588	This study
SP150104	Duck intestines	Zigong city, Sichuan province	2015	O1.1	ST4479	KY574589	This study
T150072	Faece of healthy butcher	Zigong city, Sichuan province	2015	O6	ST4619	KY574598	This study
T150248	Faece of healthy butcher	Zigong city, Sichuan province	2015	O5	ST4619	KY574599	This study
T150298	Faece of healthy butcher	Zigong city, Sichuan province	2015	O5	ST4637	KY574600	This study
SH13EC413	Faece of diarrhoeal patient	Shanghai	2013	O6	ST4488	KY596024	This study
SP150175	Duck intestines	Zigong city, Sichuan province	2015	O1.1	ST4606	KY574590	This study
SP150183	Duck intestines	Zigong city, Sichuan province	2015	O1.1	ST4606	KY574591	This study
SP150185	Duck intestines	Zigong city, Sichuan province	2015	O6	ST4637	KY596023	This study
SP150193	Chicken intestines	Zigong city, Sichuan province	2015	O1.1	ST4479	KY574592	This study
SP150242	Duck intestines	Zigong city, Sichuan province	2015	O1.8	ST4606	KY574593	This study
SP150249	Duck intestines	Zigong city, Sichuan province	2015	O1.9	ST4606	KY574594	This study
SP150253	Duck intestines	Zigong city, Sichuan province	2015	O1.9	ST4606	KY574595	This study
SP150265	Duck intestines	Zigong city, Sichuan province	2015	O1.10	ST4606	KY574596	This study
SP150270	Duck intestines	Zigong city, Sichuan province	2015	O3	ST4633	KY574597	This study
LX16057	Chicken intestines	Luzhou city, Sichuan province	2016	O1	ST4479		This study
LX160195	Chicken intestines	Luzhou city, Sichuan province	2016	O1	ST4479		This study
LX160135	Chicken intestines	Luzhou city, Sichuan province	2016	O1	ST4606		This study
LX16061	Chicken intestines	Luzhou city, Sichuan province	2016	O1	ST4479		This study
LX16053	Chicken intestines	Luzhou city, Sichuan province	2016	O1	ST4479		This study
LX160190	Chicken intestines	Luzhou city, Sichuan province	2016	O1	ST4479		This study
LX160154	Chicken intestines	Luzhou city, Sichuan province	2016	O1	ST4606		This study
LX16058	Chicken intestines	Luzhou city, Sichuan province	2016	O1	ST4479		This study
LX160100	Chicken intestines	Luzhou city, Sichuan province	2016	O1	ST4606		This study
LX16059	Chicken intestines	Luzhou city, Sichuan province	2016	O2	New		This study
LX16054	Chicken intestines	Luzhou city, Sichuan province	2016	O2	ST3762		This study
LX160162	Chicken intestines	Luzhou city, Sichuan province	2016	O4	ST4638		This study

### Sequencing and bioinformatics analysis

Forty-two strains were chosen for whole genome sequencing (WGS) based on the MLST results. For each strain, a library was constructed (500–2,000 bp) and then sequenced on an Illumina Hiseq 4,000 system (Illumina, San Diego, CA, USA) to produce 150 bp paired-end reads, which were then assembled into scaffolds using the program SOAP *de novo* (Release 1.04, http://soap.genomics.org.cn/soapdenovo.html). Open reading frames (ORFs) were identified and annotated using the Artemis program (www.sanger.ac.uk) and homology searches against several databases including GenBank (www.ncbi.nlm.nih.gov/GenBank), the Clusters of Orthologous Groups (COG; www.ncbi.nlm.nih.gov/COG/), and Pfam (pfam.sanger.ac.uk) protein motif databases (Altschul et al., 1997; Tatusov et al., 2001; Bateman et al., 2002). Each O-AGC between the *galF* and *gnd* genes was extracted from the draft genome sequence. Based on these O-AGC sequences, another 10 O-AGCs were sequenced by primer walking PCR. The TMHMM (v2.0) analysis program (http://www.cbs.dtu.dk/services/TMHMM/) was used to identify potential transmembrane segments from the amino acid sequences. The Artemis comparison tool (ACT) (Carver et al., 2005) was used to visualize the data.

### Preparation of specific antisera

Based on the typing result of strains SP140089, SP150020, SP140724, D140513, T150248, T150072, and ZG141049 were initially used as standard antigen strains to produce antisera. Three New Zealand white rabbits (female, 1.5 to 2 kg body weight) were immunized intravenously with heat-killed (100°C, 2 h) cells four times with the same doses (2.5 × 10^10^ CFU) for each strain. The second immunization booster was performed 14 days after the first immunization. The third and fourth immunization boosters were performed 5 days and 10 days after the second immunization, respectively. Serum was obtained 5 days after the last immunization. Prepared serum was used to test all *E. albertii* strains in this study by slide agglutination and the strain was heat-killed in 100°C for an hour before the agglutination test. Visual agglutination apparent within 20 s was recorded as a positive result. The antiserum that agglutinated all corresponding serotype strains but did not agglutinate any other strains from other serotype groups was referred to as specific antiserum for the corresponding serotype.

### Development of the high throughput xTAG luminex detection assay

MagPlex –xTAG Microspheres (superparamagnetic beads in 6.5 microns diameter) precoupled with a 24- base oligonucleotide “anti-TAG” sequence were used in the assay. Sequences and working concentration of the primers used to amplify *E. albertii* specific gene *lysP* (Hyma et al., 2005) and the serotype-specific *wzy* gene were listed in Table 2. The primers were designed based on the principles described in previous study (Bai et al., 2015). Briefly, the lengths of the primers were between 22 and 26 oligonucleotides, their melting temperatures were between 49 and 52°C, and the amplification size ranged between 100 and 500 base pairs. In order to conjugate with MagPlex –xTAG Microspheres, a corresponding 24- base oligonucleotide “TAG” sequence was added at the 5′ terminus of each upstream primer. To facilitate the interaction between MagPlex –xTAG Microspheres and amplified productions, a spacer was made by incorporating a 12-carbon amine containing group between “TAG” sequence and primer. Each downstream primer was biotinylated at the 5′ terminus. The products were amplified using cycling parameters at 94°C for 5 min; 30 cycles of 94°C for 30 s, 56°C for 30 s, and 72°C for 30 s, followed by a final elongation step at 72°C for 10 min. The threshold of the detection limit was determined by using serially diluted DNA from a representative strain of each serotype. Pure genomic templates from 113 stocked strains used in our previous study (Wang Y. et al., 2016) were also used to determine the specificity of the system in the study. Two independent experiments were performed to establish the sensitivity and specificity of the system.

**Table 2:** Serotype-specific primers used in this study.

**Serotype**	**No. of beads coupled with “anti-TAG” sequences**	**Sequence (5′–3′)**	**Working concentration (μM)**	**Sensitivity (pg)**	**PCR product size (bp)**
O1	39	ACAAATATCTAACTACTATCACAA**–12C–**TCCAGTCTTCTTTCGGAATTTT	0.3	0.5	107
		^*^AAGTTCATGCGTGGAAAAATAC			
O2	42	CACTACACATTTATCATAACAAAT**–12C–**ATAGCGGGGTATTTGGATTTAC	0.3	1	232
		^*^TACAACCGACAAGAAGAAACAA			
O3	43	AACTTTCTCTCTCTATTCTTATTT**–12C–**ATCTTCACGCTCTTTTTACTGA	0.3	1	275
		^*^TATAACCCTGCAATTACCGAAG			
O4	44	TCATCACTTTCTTTACTTTACATT**–12C–**TTACTGCGTTGATGAAAGTTTG	0.3	0.5	102
		^*^CGCAATAACGGTAAACAAAGAA			
O5	45	TACACAATATTCATCATAACTAAC**–12C–**GCGGGGATTATTACTTTTAGGT	0.3	10	271
		^*^CTCCATATCGCAGGTCAAAATA			
O6	46	TTAAACAATCTACTATTCAATCAC**–12C–**GTGGGTGAAAGTAAGGTCAATA	0.3	1	212
		^*^TCTGAAAATGGGATGAATGACA			
O7	47	TCTCTTTAAACACATTCAACAATA**–12C–**AGATATAACGTCGGCATTGATT	0.3	10	250
		^*^ATAGCAACCCAACCACATAAAA			
All (*lysP*)	48	AATCAACACACAATAACATTCATA**–12C–**GGGCGCTGCTTTCATATATTCTT	0.1	1	252
		^*^TCCAGATCCAACCGGGAGTATCAGGA			

xTAG sequences and the 12-carbon amine containing group are indicated by underline and bold text, respectively.

* *Indicates reverse primer is biotinylated at the 5′ terminus*.

### Ethics statement

This study was reviewed and approved by the ethics committee of the National Institute for Communicable Disease Control and Prevention, Chinese Center for Disease Control and Prevention. The rights and the welfare of the rabbits used in the study were adequately protected. All necessary steps were taken to minimize suffering and distress to the rabbits in these studies.

## Results

### Grouping and general features of the O-AGCs

On the basis of sequences and genetic structures of the entire O-AGC regions, the O-AGCs from 52 strains were placed into seven groups (denoted O1–O7) where O1 (*n* = 17) was the most prevalent, followed by O4 (*n* = 9), O2 (*n* = 8), O3 (*n* = 6), O5 (*n* = 5), O6 (*n* = 4), and O7 (*n* = 3). In our previous studies, we dissected the chemical structures of the O-specific polysaccharides (OPSs) of *E. albertii* O1, O3, O4, O6, and O7 (Naumenko et al., 2017; Zheng et al., 2017). The predicted gene functions of O-AGCs were consistent with their OPS structures. The data indicated that O-AGCs extracted are responsible for the O-antigen synthesis of *E. albertii*.

All O-AGCs carried the *wzx* and *wzy* genes. The DNA sequence identities of the *wzy* and *wzx* genes were >99.9% within the same serotype group and <8% among different serotype groups. Genes coding for enzymes involved in the synthesis of sugars forming the O subunit and glycosyltransferases were found in each serotype group.

**Figure 1:**
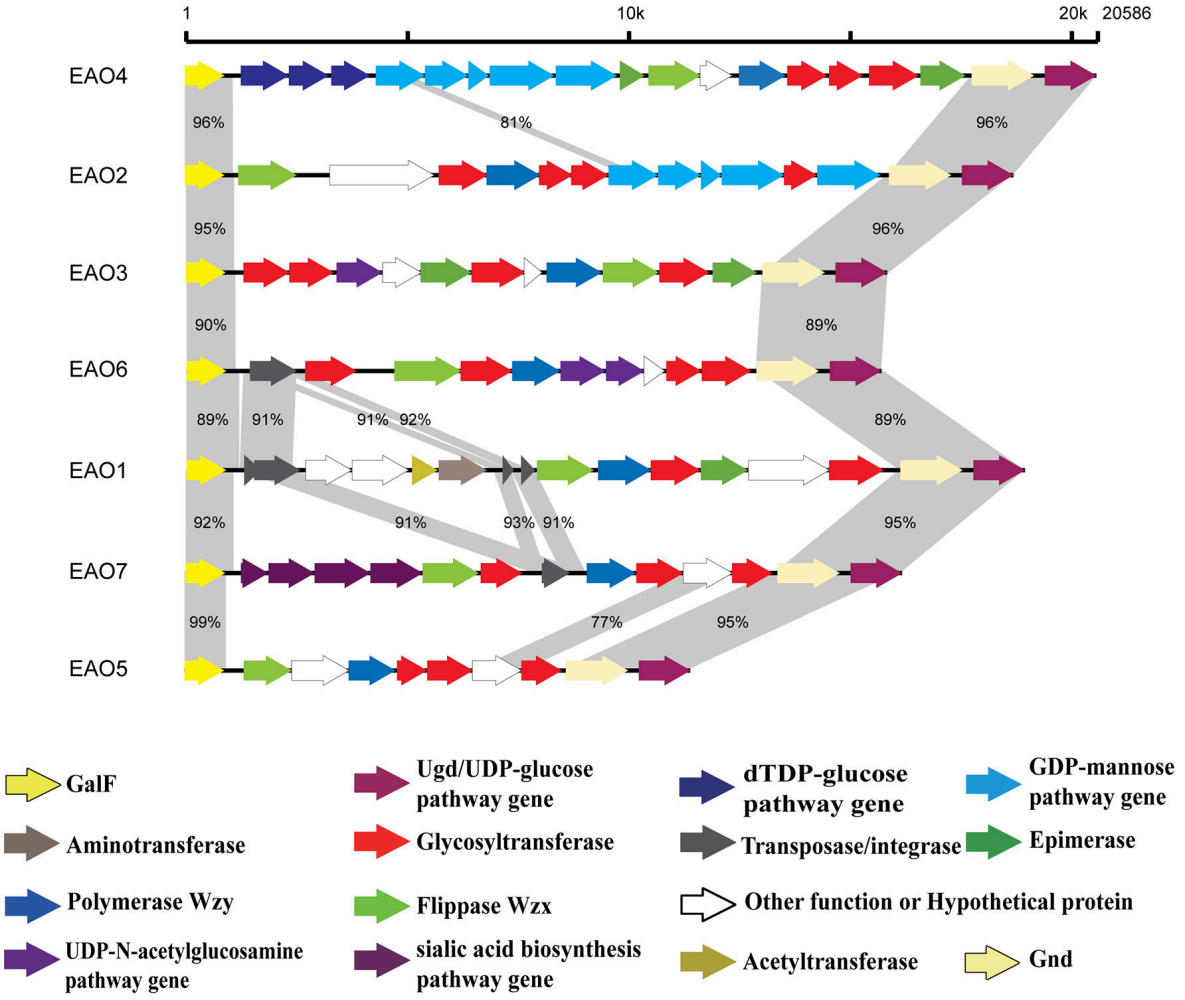
The seven *E. albertii* O-AGCs identified in this study. The corresponding CDSs are colored as indicated. The similarities higher than 50% were shadowed.

Significant differences among the seven groups were also observed where the size ranged from 7.2 kb (O5, including 7 genes) to 16.4 kb (O4, including 16 genes) and the G+C content of seven O-AGCs ranged from 29.2% (O5) to 38.9% (O4). Simple insertions of transposase genes were found in *E. albertii* O1, O6, and O7 without any gene disruption (Figure 1). Genetic heterogeneity was only found within *E. albertii* O1-AGC. Within the six other O-AGC groups, we observed high sequence conservation (>99% DNA sequence identity).

Within *E. albertii* O1-AGC, all strains harbored a transposase gene in the 5′ region. The tandem repeat number of “CTCTG” in the transposase gene was different between the strains (eight types of tandem repeat were found from 11 copies to 73 copies). Meanwhile, two types of transposase gene were found in central regions of *E. albertii* O1-AGC. Nineteen *E. albertii* O1 strains were assigned into 10 subtypes based on the variable sequence and organization of transposase genes, named *E. albertii* O1.1-AGC to O1.10-AGC. *E. albertii* O1.1-AGC (*n* = 5) was the dominant subtype (Table 1).

### MLST of *E. albertii*

Thirteen sequence types (STs) were found in 52 strains. Eleven of these were reported previously (Wang H. et al., 2016). *E. albertii* O1, O2, O4, O5, and O7 contained multiple STs (Table 1). All strains with identical STs were clustered into the same serotype group.

### Comparison of O-AGCs between *E. albertii* and other species

In our previous studies, we have found that the O-antigen of *E. albertii* O1, O3, O4, O6, and O7 is structurally and genetically related to the O-antigens of other species (Naumenko et al., 2017; Zheng et al., 2017). In the study, we compared the O-AGCs of *E. albertii* O2 and O5 to those of other species.

*orf8* to *orf13* in O-AGC of *E. albertii* O2 showed 91% similarity to genes (*orf10* to *orf15*) in O-AGC of *E. coli* O156 (GenBank accession no. AB812065). Five from them (*orf8-11* and *orf13*) are homologs of *gmd, fcl, gmm, manB* and *manC* genes. *manB and manC* genes were involved in the synthesis of GDP-d-Man from Fru-6-P. *gmd* and *fcl genes* are responsible for the synthesis of GDP-l-Fuc from GDP-d-Man. The protein encoded by *gmm* gene, which catalyzes hydrolysis of GDP-d-Man to yield GDP and d-Man, has been suggested to participate in the regulation of cell wall biosynthesis by influencing the concentration of GDP-d-Man in the cell wall (Perepelov et al., 2015; Duan et al., 2016). *orf12* of *E. albertii* O2 is homologs of *orf14* (glycosyltransferase) of *E. coli* O156. *orf14* of *E. coli* O156 named *wefY* is responsible for the formation of α-l-Fucp-(1 → 3)-d-GlcpNAc linkage (Duan et al., 2016). It is reasonable to propose that *E. albertii* O2 has the similar linkage.

Two ORFs of *E. albertii* O5 (*orf6-7*) showed 80% and 76% identities in amino acid to the *glf* and *wfeT* genes of *Shigella dysenteriae* type 3 O-antigen gene cluster (GenBank: EU296415), respectively. *E. albertii* O5 may share the OPS main chain synthesized by *glf* and *wfeT* genes with *S. dysenteriae* type 3 (Liu et al., 2008).

### Distribution of seven O-AGCs in published *E. albertii* sequences

Four complete genome sequences and 25 draft sequences of *E. albertii* strains have been reported recently (Fiedoruk et al., 2014; Ooka et al., 2015). Meanwhile, two additional genome draft sequences (BBMY00000000 and NZ_CH991859.1) were available in the NCBI database. Analysis of their O-AGCs was performed and six types of O-AGCs reported in the present study were also found in 15 previously published genomes. Contrary to Chinese strains, *E. albertii* O7 (*n* = 4) was the most prevalent amongst these, followed by O2 (*n* = 3), O1 (*n* = 2), O3 (*n* = 2), O4 (*n* = 2), and O5 (*n* = 2) (Table S1). Another 16 published genomes harbored novel O-AGCs, which were placed into 13 groups. All 16 O-AGCs possessed similar characteristics to those of *E. albertii* O1 -7 in that: (i) located in a fixed region between *galF* and *gnd*; (ii) carried the *wzx* and *wzy* genes. It is noteworthy that *wzy* was serotype-specific gene of 13 O-AGCs.

The size ranged from 8.1 kb (CB9791, including 8 genes) to 16.4 kb (NIAH_Bird_23, including 14 genes) and the G+C content ranged from 31.5% (K7394) to 39.1% (CB9791). Thirteen of them were found homologies in O-AGCs of *E. coli*, including O41, O49, O58, O65, O115, O128, O130, O152, O182, and O184 (Table S1). Three of them were not found homologies in any O-AGCs of other species.

### Establishment of *E. albertii* O serogroups

Seven antisera were ultimately selected for the current *E. albertii* serotyping scheme, and all 52 tested *E. albertii* strains were clearly assigned to one of these seven serotypes. The agglutination results of 52 strains were completely consistent with their O-AGCs grouping results. All antisera were specific for their homologous strains. In general, homologous titers were high, varying from 640 to 1280 (Table 3). An additional 12 strains isolated in 2016 were also typed using the seven antisera. Nine of these were typed as *E. albertii* O1, while the others were typed as *E. albertii* O2 (2) and *E. albertii* O4 (1), respectively (Table 1).

**Table 3:** Agglutination of *E. albertii* antisera^*^.

**Antiserum type**	**Agglutination titer to serotype strains**						
	**O1 (SP140089)**	**O2 (SP150020)**	**O3 (SP140724)**	**O4 (D140513)**	**O5 (T150248)**	**O6 (T150072)**	**O7 (ZG141049)**
O1	1,280						
O2		1,280					
O3			1,280			<20	
O4				1,280			
O5			<20		1,280		
O6						1,280	
O7						<20	640

* *Agglutination titers lower than 10 are not shown*.

### Development and evaluation of a high throughput xTAG luminex assay to simultaneously detect seven O-AGCs

Specific detection was based on the unique sequence of *wzy* for each O-AGC (Table 2). The *wzy* gene was amplified in a multiplex PCR format. The detection limit for the seven O-AGCs varied from 0.5 to 10 pg of purified DNA per reaction.

The performance of this system was tested on 64 strains used in the study. Cross- and non-specific amplification between sequences was not observed. All 64 strains were correctly designated to corresponding serotype groups, which were completely consistent with their agglutination test results.

## Discussion

Serotyping remains the “gold standard” for identifying and monitoring organisms. The chemical composition and structure of the O antigen show high levels of variation even within a single species revealing it to be serologically diverse. Prior to the present study, little was known about the distribution and diversity of O-AGCs in *E. albertii*. Combination of chemical structures of the O-specific polysaccharides (OPSs) of *E. albertii* (Naumenko et al., 2017; Zheng et al., 2017) and sequence analysis, serotyping scheme in the present study, we defined the characteristics of O-AGCs in *E. albertii*: (i) Similar to *E. coli*, all O-AGCs of *E. albertii* were located in a fixed region of the genome between *galF* and *gnd*. (ii) High diversity among different O-AGCs group was observed. (iii) O-antigens of *E. albertii* were synthesized by the Wzx/Wzy-dependent pathway. The Wzx/Wzy-dependent assembly pathway is conserved in a wide range of both Gram-negative and Gram-positive bacteria, and is encoded in dedicated gene clusters. Within 185 well identified O-AGCs in *E. coli*, 174 of these were synthesized by the Wzx/Wzy pathway (Iguchi et al., 2015). Within 90 serotypes of *Streptococcus pneumoniae*, 88 of these were synthesized by the Wzx/Wzy pathway (Bentley et al., 2006).

In this study, we found and named seven serotypes as *E. albertii* O1–O7 in Chinese strains. Amongst these, the O1 serotype comprised approximately 40% (26/64) of all the strains, which was the most dominant serotype. Even though *E. albertii* O1 was the dominant serotype identified in this study, it was not found in strains from diarrheal patients in the current and previous studies (Fiedoruk et al., 2014; Ooka et al., 2015) (Table 1 and Table S1). Further studies are needed to understand the relationship between serotype and pathogenic potential. Different from Chinese strains, 19 types of O-AGCs were found in 31 public genomes of *E. albertii*, which were composed of 6 serotypes reported in the present study and 13 different O-AGCs. This may suggest that host specificity and ecological environment may contribute to the serotype diversity of strains between China and other countries.

To date, 20 O-AGCs of *E. albertii* were identified. It is relatively lower compared to 185 O serogroups of *E. coli* (Iguchi et al., 2015), 54 serotypes of *Shigella* spp. (Muthuirulandi Sethuvel et al., 2017) and more than 200 serotypes of *Vibrio cholerae* (Stine and Morris, 2014). We cannot rule out the possibility that more serotypes may be found with additional testing of *E. albertii* strains.

In this study, we performed an O-antigen serotyping scheme for *E. albertii* based on specific antisera against seven O-antigens, but the conventional serotyping method using the agglutination test with serotype-specific antisera is laborious, time-consuming and expensive. High-throughput molecular serotyping methods allow for simultaneous detection of multiple nucleic acid sequences in a single reaction, and can greatly reduce the time, cost, and work. These technologies have become attractive alternatives to conventional serotyping methods. mPCR coupled to Luminex xTAG technology-based detection provides a clear and attractive approach for multiplex analysis. The low conservation between *wzy* genes of different serotypes means *wzy* gene is an excellent molecular marker for molecular serotyping. In present study, a high throughput xTAG Luminex assay using unique sequence of *wzy* for each serotype to simultaneously detect seven O-AGCs was developed. All the tested strains were accurately typed into seven O-AGCs which were completely consistent with their seroagglutination results. The detection system can be completed in 40 min post-PCR amplification. The limitation of the system is that only seven serotypes revealed in the study can be detected. Thirteen O-AGCs present in public genome of *E. albertii* (Table S1) were not added in the system for lacking strains to evaluate the system. However, the system has great potential to increase the multiplicity in a single reaction.

Comparing to whole genome, the pretty low G+C content of 20 O-AGCs suggest that they may have originated from a different species. *E. albertii* has recently been recognized as a close relative of *E. coli* (Ooka et al., 2015). It is noteworthy that 12 O-AGCs of *E. albertii* were found homologies in O-AGCs of *E. coli*. Additionally, many genes present in O-AGCs of *E. albertii* were also widely distributed in O-AGCs of *E. coli*. Moreover, all strains of *E. albertii* O3 and O6 were agglutinated with *E. coli* O181 and O3 serum, respectively. It is noteworthy that identical O-AGCs among different species have also been reported in previous studies (Sugiyama et al., 1997; Cheng et al., 2006; Feng et al., 2007). Meanwhile, the *E. albertii* strains with identical O-antigens were isolated from diverse sources and belonged to different sequence types. The finding suggested the O-AGCs can also readily spread among *E. albertii* strains, even among *Enterobacteriaceae*. Further studies are needed to understand the ability of this organism to spread and cause disease.

In conclusion, our data revealed the highly genetic diversity of O-AGCs in *E. albertii* and that *E. albertii* O1 was the dominant serotype. Our study provided valuable serotyping methods for the epidemiological study of this newly emerging enteric pathogen.

## Author contributions

HZ, HJ, JX, and YX designed the project; HW, QL, XL, LZ, NZ, GY, and ZZ carried out the sampling work; HW, HZ, YXu, JW, PD, and XqL carried out the experiments and generated data; HZ and YX analyzed data and drafted the manuscript. All authors have read and approved the final version of the manuscript.

### Conflict of interest statement

The authors declare that the research was conducted in the absence of any commercial or financial relationships that could be construed as a potential conflict of interest.
